# Validity of oral fluid test for Delta-9-tetrahydrocannabinol in drivers using the 2013 National Roadside Survey Data

**DOI:** 10.1186/s40621-018-0134-2

**Published:** 2018-02-19

**Authors:** Huiyan Jin, Sharifa Z. Williams, Stanford T. Chihuri, Guohua Li, Qixuan Chen

**Affiliations:** 1 Department of Biostatistics, Columbia University Mailman School of Public Health, 722 West 168th Street, New York, NY 10032 USA; 2 Department of Anesthesiology, Columbia University College of Physicians and Surgeons, 622 West 168th Street, New York, NY 10032 USA; 3 Department of Epidemiology, Columbia University Mailman School of Public Health, 722 West 168th Street, New York, NY 10032 USA

**Keywords:** Blood samples, Cannabis, Delta 9-tetrahydrocannabinol, Driving under the influence, Marijuana, Oral fluid samples

## Abstract

**Background:**

Driving under the influence of marijuana is a serious traffic safety concern in the United States. Delta 9-tetrahydrocannabinol (THC) is the main active compound in marijuana. Although blood THC testing is a more accurate measure of THC-induced impairment, measuring THC in oral fluid is a less intrusive and less costly method of testing.

**Methods:**

We examined whether the oral fluid THC test can be used as a valid alternative to the blood THC test using a sensitivity and specificity analysis and a logistic regression, and estimate the quantitative relationship between oral fluid THC concentration and blood THC concentration using a correlation analysis and a linear regression on the log-transformed THC concentrations. We used data from 4596 drivers who participated in the 2013 National Roadside Survey of Alcohol and Drug Use by Drivers and for whom THC testing results from both oral fluid and whole blood samples were available.

**Results:**

Overall, 8.9% and 9.4% of the participants tested positive for THC in oral fluid and whole blood samples, respectively. Using blood test as the reference criterion, oral fluid test for THC positivity showed a sensitivity of 79.4% (95% CI: 75.2%, 83.1%) and a specificity of 98.3% (95% CI: 97.9%, 98.7%). The log-transformed oral fluid THC concentration accounted for about 29% of the variation in the log-transformed blood THC concentration. That is, there is still 71% of the variation in the log-transformed blood THC concentration unexplained by the log-transformed oral fluid THC concentration. Back-transforming to the original scale, we estimated that each 10% increase in the oral fluid THC concentration was associated with a 2.4% (95% CI: 2.1%, 2.8%) increase in the blood THC concentration.

**Conclusions:**

The oral fluid test is a highly valid method for detecting the presence of THC in the blood but cannot be used to accurately measure the blood THC concentration.

## Background

Marijuana is a commonly used drug in the United States. In 2015, there were an estimated 22.2 million Americans aged 12 years or older reporting current marijuana use and 19.8% (about one in every five) young adults aged 18 to 25 years were current users (Center for Behavioral Health Statistics and Quality 2016). Experimental and epidemiological studies showed that recent marijuana use is associated with decreased driving performance—e.g., decreased reaction time, reduced lateral control, driving slower speeds, and impairment of psychomotor skills—and thus increased motor vehicle crash risk (Brady and Li 2013; Brady and Li 2014; Hartman and Huestis 2013; Chihuri et al. 2017). Driving under the influence (DUI) of marijuana is of increasing concern in the United States (Berning et al. 2015; Li et al. 2016; Davis et al. 2016; Johnson et al. 2012; Edwards et al. 2017). The annual number of fatal automobile crashes attributable to impaired driving has been on the rise and marijuana is the most frequently detected non-alcohol drug in drivers in the United States (Brady and Li 2013; Brady and Li 2014; Romano and Voas 2011; Tefft et al. 2016; Downey et al. 2013; Hartman et al. 2015; Lennéa et al. 2010; Li et al. 2012). From 1999 to 2010, the prevalence of marijuana detected among drivers involved in fatal car crashes tripled from 4.2% to 12.2% (Brady and Li 2014).

Delta 9-tetrahydrocannabinol (THC) is the primary psychoactive compound in marijuana and its presence in blood samples is viewed as an indicator of recent (1–8 h after smoking or oral intake) marijuana use (Karschner et al. 2012; Hartman et al. 2016; Jones et al. 2008). Because blood THC test is a more accurate measure of THC-induced impairment (Hartman et al. 2016; Grotenhermen et al. 2007), many US states use blood THC concentrations as the gold standard for determining marijuana-related DUI (Center for Behavioral Health Statistics and Quality 2016; Berning et al. 2015). Different cut-off points of blood THC concentrations have been proposed for establishing per se laws, which make operating a motor vehicle a criminal offense for an individual if he or she has a specific amount of drug or metabolite in his or her body. This threshold concentration is a legal limit, and exceeding this threshold serves as proof of legal impairment (Grotenhermen et al. 2007; Wong et al. 2014; National Conference of State Legislatures 2017). Currently, 20 states have established per se limit laws on marijuana-impaired driving, with four states using 5 nanograms per milliliter (ng/ml) as the threshold for THC and the others having zero tolerance (National Conference of State Legislatures 2017).

Compared to collecting whole blood samples, measuring THC in oral fluid is a less intrusive and less costly method of testing (Grotenhermen et al. 2007; Bosker and Huestis 2009; Drummer 2005; Drummer 2006). Although no accurate predictive function has been established between the oral fluid THC concentration and the whole blood THC concentration, studies have showed a correlation between these two test results (Lee and Huestis 2014). Hence oral fluid THC testing has potential utility as a standard screening tool for detecting marijuana-related DUI (Gjerde et al. 2014; Langel et al. 2014; Gjerde et al. 2015; Edwards et al. 2017). In this study, we assess the validity of oral fluid THC test versus blood THC test using sensitivity and specificity analysis and a logistic regression, and estimate the quantitative relationship between the oral fluid THC concentration and the blood THC concentration using a correlation analysis and a linear regression on the log-transformed THC concentrations.

## Methods

### Data source

Data for this study came from the 2013 National Roadside Survey of Alcohol and Drug Use by Drivers (NRS) conducted by the National Highway Traffic Safety Administration, the National Institute on Drug Abuse, and the Insurance Institute for Highway Safety. The aim of the NRS was to estimate the prevalence of driving under the influence of alcohol and/or drugs in the United States (Berning et al. 2015). The first NRS was conducted in 1973; since then, it has been conducted in 1986, 1996, 2007 and 2013. The 2013 NRS was a national field survey based on voluntary and anonymous random stops of non-commercial drivers at 300 locations across the 48 contiguous states (Kelley-Baker et al. 2016). The sample was selected using multistage sampling method and only verbally consented drivers were included in the survey. Data were collected from drivers in 60 locations during the hours of 9:30 AM to 11:30 AM and 1:30 PM to 3:30 PM on Fridays and another 240 locations 10 PM to 12 AM and 1 AM to 3 AM on Fridays and Saturdays from June 7, 2013 through March 30, 2014. Detailed information on the 2013 NRS methodology can be found elsewhere (Kelley-Baker et al. 2016).

The survey included questions on driver demographic characteristics such as age and race, drinking and drug use habits such as time of last marijuana use, trip information including trip origin and destination, seatbelt usage, and vehicle information such as number of passengers and vehicle type. All information was collected from the drivers via electronic tablet except for gender, which was recorded via officer observation. In addition, breath alcohol, oral fluid alcohol, and oral fluid drug concentration tests were administered and whole blood specimens were collected during the survey process (Kelley-Baker et al. 2016). Among 11,100 eligible drivers, 8802 (79.3%) voluntarily participated in the 2013 NRS, and 4669 (42.1%) consented to provide both blood and oral fluid samples for drug and alcohol testing. For this study, we used the data from 4596 drivers aged 16 years and older with both blood and oral fluid THC concentration test results. Compared to all the drivers who participated in the 2013 NRS, these 4596 drivers had a similar age distribution and proportion of females, but included fewer whites (59.1% vs. 62.0%) and were more likely to report either never having used marijuana or the last time of use to be beyond a year (79.4% vs. 69.5%).

### Measures of interest

In the 2013 NRS, 1 ml (ml) of oral fluid sample and 10 ml of whole blood specimen were collected from each consented driver. The presence of THC in oral fluid and whole blood samples were first screened by Enzyme-linked Immunosorbent Assay (ELISA) (Moore et al. 2006). The minimum detectable screening concentration of THC in oral fluid, as measured by ELISA, was 4 ng/ml. Samples screened as having THC concentrations ≥4 ng/ml were further tested using liquid/gas chromatography and mass spectrometry (LC/MS/MS) technology with a minimum detectable THC concentration of 2 ng/ml in oral fluid. A similar screening and confirmation process was carried out for measuring THC concentrations in whole blood samples. The minimum detectable concentration of THC in blood was 10 ng/ml using the ELISA screening test and 1 ng/ml using the LC/MS/MS confirmation test. Results from the LC/MS/MS test were recorded as the final results for both oral fluid and blood samples (Kelley-Baker et al. 2016).

### Defining positivity of THC in blood and oral fluid samples

We defined a blood THC test as positive if the THC concentration was greater than 0 ng/ml (or ≥ 1 ng/ml) in the whole blood sample for two reasons. First, it is the minimum detectable concentration of THC in the whole blood. Second, it is the cutoff point for establishing per se laws by many states (National Conference of State Legislatures 2017). This cut-off was consistent with the NRS methodology (Kelley-Baker et al. 2016). To define positivity for oral fluid THC test that best predicted positivity of THC in blood, we dichotomized the oral fluid THC concentrations using a series of cut-offs greater than or equal to the minimum detectable concentration (≥ 2 ng/ml, ≥ 3 ng/ml, etc.). We conducted a series of logistic regression of the binary blood THC test (positive vs. negative) on the binary oral fluid THC test defined by each cut-off value. Each logistic regression model controlled for driver age (16–20 years, 21–34 years, 35–54 years, and 55 years or older), gender (female or male), race (white, black, or other), time of last marijuana use (beyond a year/never, over a month, within the past month to 2 days, or in the past 24 h), and blood alcohol concentration (BAC; 0 mg/dl, 1–39 mg/dl, and 40 or more mg/dl). We used a random sample with 80% of the 2013 NRS data to build logistic regression models and the remaining 20% to calculate accuracy defined as the proportion of true positive or true negative cases and area under receiver operating characteristic curve (AUC) (Robin et al. 2011). Higher values of accuracy and AUC indicate better predictive ability of the dichotomized oral fluid THC concentrations at the selected cut-off in distinguishing between positive and negative blood THC tests. The highest accuracy (0.966) was found in the cut-off values of ≥ 2 ng/ml and ≥ 3 ng/ml for oral fluid test. Although the cut-off value of ≥ 3 ng/ml yielded a slightly greater AUC (0.865) than the cut-off value of ≥ 2 ng/ml (0.863), we defined positivity of THC in oral fluid using ≥ 2 ng/ml because it was the minimum detectable THC concentration (i.e., same as using > 0 ng/ml) and was consistent with the NRS methodology (Kelley-Baker et al. 2016).

### Statistical analysis

We first calculated positive rates of THC in both blood and oral fluid samples and computed sensitivity and specificity of the oral fluid THC test in predicting the positivity of blood THC. The analysis was conducted using the whole sample and then stratified by driver characteristics.

We then fit three logistic regression models of the positivity in blood THC on oral fluid THC, which was included in the model using both a binary covariate (> 0 ng/ml vs. = 0 ng/ml) and the natural-log of the continuous THC concentration. For samples with 0 ng/ml oral fluid THC concentrations, 0.5 ng/ml was used to enable natural-log transformation. The first model assessed the crude association. The second model adjusted for BAC and time of last marijuana use. The third model further controlled for demographic characteristics of driver age, gender, and race. We tested the interaction terms between BAC and oral fluid THC test, but the interaction terms were excluded from the final models because they were not statistically significant.

We also examined the association between the continuous blood THC concentration and the continuous oral fluid THC concentration among drivers with positive THC in either blood or oral fluid. We calculated Pearson correlation coefficient between these two concentrations in both the original and natural-log transformed scales. We then built linear regression models of the natural-log transformed blood THC concentration on the natural-log transformed oral fluid THC concentration. Similar to the logistic regression, we used a value of 0.5 ng/ml for 0 ng/ml THC concentrations to enable natural-log transformation and considered both the unadjusted and the two adjusted models.

Finally, we conducted two sensitivity analyses. In the first sensitivity analysis, we multiply imputed the missing data on gender and time of last use of marijuana using the multiple imputation by chained equations algorithm to create complete data for the covariates in the adjusted models (Van Buuren and Groothuis-Oudshoorn 2011). The second sensitivity analysis accounted for complex survey design in the 2013 NRS survey. Analyses were conducted using R version 3.2.4 (R Development Core Team, Vienna, Austria).

## Results

**Fig. 1:**
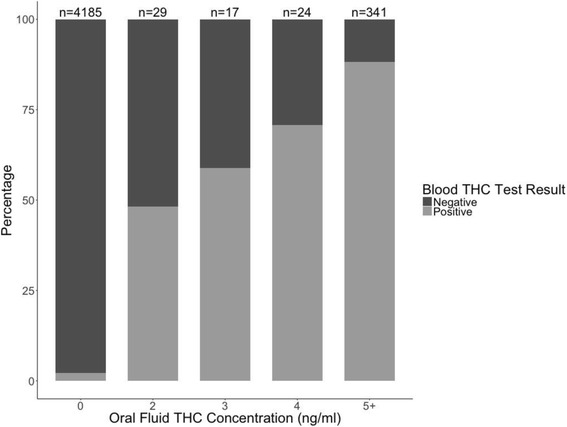
Percentages of Drivers with Positive and Negative Blood THC at Different Levels of Oral Fluid THC Concentration using the Data of Drivers Aged 16 Years and Older with both Blood and Oral Fluid THC Test Results in the 2013 NRS (*n* = 4596)

Figure 1 shows the percentages of positive and negative blood THC tests at different oral fluid THC concentrations (0, 2, 3, 4, and ≥5 ng/ml). Most of the drivers with fluid THC concentrations under the minimum detection limit (< 2 ng/ml or = 0 ng/ml) were negative in the blood THC tests. The percentage of positivity in blood THC increased as the concentration of oral fluid THC increased.

Table 1 provides the positive rates of THC in blood and oral fluid samples as well as the sensitivity and specificity of the positivity of oral fluid THC in predicting the positivity of blood THC. Overall, 9.4 and 8.9% of the samples tested positive for THC in blood and oral fluid, respectively, yielding a sensitivity of 79.4% with a 95% confidence interval (CI) (75.2%, 83.1%) and a specificity of 98.3% (95% CI: 97.9%, 98.7%) for the oral fluid test in predicting positivity in blood THC. The oral fluid test had the highest sensitivity among drivers with positive BAC but lowest sensitivity among drivers older than 55 years of age. The specificity was higher than 90% in all groups, except drivers with the last use of marijuana in the past 24 h (66.7%, 95% CI: 52.1%, 79.2%).

**Table 1:** Percentages of Drivers with THC Positivity in Blood and Oral Fluid and Sensitivity-Specificity Analysis of the Oral Fluid Test using the Blood Test as the Reference Criterion. The Analyses were performed for All Drivers and Stratified by Driver’s Characteristics

	N	Positivity in blood THC N (%)	Positivity in oral fluid THC N (%)	Sensitivity % (95% CI)	Specificity % (95% CI)
All	4596	431 (9.4)	411 (8.9)	79.4 (75.2, 83.1)	98.3 (97.9, 98.7)
Age (years)					
16–20	479	76 (15.9)	75 (15.7)	81.6 (71.0, 89.6)	96.8 (94.6, 98.3)
21–34	1909	241 (12.6)	233 (12.2)	80.1 (74.5, 84.9)	97.6 (96.8, 98.3)
35–54	1491	92 (6.2)	88 (5.9)	81.5 (72.1, 88.9)	99.1 (98.4, 99.5)
≥ 55	717	22 (3.1)	15 (2.1)	54.6 (32.2, 75.6)	99.6 (98.7, 99.9)
Gender ^a^					
Female	1980	143 (7.2)	128 (6.5)	74.1 (66.1, 81.1)	98.8 (98.2, 99.3)
Male	2597	282 (10.9)	279 (10.7)	82.3 (77.3, 86.5)	98.0 (97.3, 98.5)
Race					
White	2718	186 (6.8)	173 (6.4)	74.7 (67.9, 80.8)	98.7 (98.1, 99.1)
Black	1015	181 (17.8)	182 (17.9)	86.7 (80.9, 91.3)	97.0 (95.6, 98.0)
Other	863	64 (7.4)	56 (6.5)	71.9 (59.2, 82.4)	98.8 (97.7, 99.4)
Last time use of marijuana ^b^					
Beyond a year/Never	3651	106 (2.9)	107 (2.9)	72.6 (63.1, 80.9)	99.2 (98.8, 99.4)
Over a month	294	51 (17.3)	50 (17.0)	84.3 (71.4, 93.0)	97.1 (94.2, 98.8)
Past month/2 days	279	98 (35.1)	87 (31.2)	77.6 (68.0, 85.4)	93.9 (89.4, 96.9)
Past 24 h	209	158 (75.6)	148 (70.8)	82.9 (76.1, 88.4)	66.7 (52.1, 79.2)
Blood alcohol concentration (ng/dl)					
0	4485	405 (9.0)	379 (8.5)	78.3 (73.9, 82.2)	98.5 (98.1, 98.8)
1–39	34	12 (35.3)	14 (41.2)	100.0 (73.5, 100)	90.9 (70.8, 98.9)
40+	77	14 (18.2)	18 (23.4)	92.9 (66.1, 99.8)	92.1 (82.4, 97.4)

^a^ 19 missing data on sex

^b^ 163 missing data on last time use of marijuana

Estimated odds ratios (ORs) and associated 95% confidence intervals from the logistic regression of the positivity in blood THC given oral fluid THC are presented in Table 2. Both the unadjusted and adjusted regression models showed that positivity in oral fluid THC was significantly associated with positivity in blood THC. After adjusting for driver BAC, last time use of marijuana and demographic characteristics, having a positive oral fluid THC test was associated with an 11-fold increase in the odds of having a positive blood THC test. The odds of positivity in blood increased as the concentration of the oral fluid THC increased, where every 10% increase in oral fluid THC was associated with an additional 6% (95% CI: 5.1%, 7.7%) increase in the odds of positive blood THC. Other covariates significantly associated with positivity in blood THC included BAC, race, and last time use of marijuana. By including BAC and last time use of marijuana, the predictive ability of the logistic regression improved with AUC increased from 0.87 (95% CI: 0.82, 0.91) to 0.95 (95% CI: 0.91, 0.98).

**Table 2:** Estimated Odds Ratios (OR) and 95% Confidence Intervals (CI) in Adjusted and Unadjusted Logistic Regression Models for THC Positivity in Blood given Oral Fluid Concentration (ng/ml), Continental United States, Nighttime Weekend and Daytime Friday, 2013–2014

	Model 1 (n = 4596)	Model 2 (*n* = 4433)	Model 3 (*n* = 4415)
	OR (95% CI)	OR (95% CI)	OR (95% CI)
Positive oral fluid test result	28.46 (13.86, 58.46)	12.10 (5.15, 28.41)	11.36 (7.32, 17.65)
Log(oral fluid THC) (log-ng/ml)	1.82 (1.48, 2.24)	1.93 (1.51, 2.47)	1.91 (1.68, 2.17)
Blood alcohol concentration (ng/dl)			
0		Ref	Ref
1–39		0.88 (0.20,3.82)	0.85 (0.39, 1.82)
40+		0.19 (0.05, 0.72)	0.19 (0.10, 0.38)
Last time used marijuana			
Over a year/Never		Ref	Ref
Over a Month		3.87 (2.26, 6.61)	3.91 (2.95, 5.16)
Past 2 days to a month		8.78 (5.40, 14.26)	8.78 (6.81, 11.33)
Past 24 Hours		25.16 (14.26, 44.41)	26.44 (19.64, 34.59)
Age (years)			
16–20			Ref
21–34			1.27 (0.96, 1.68)
35–54			1.16 (0.84, 1.59)
≥ 55			0.96 (0.63, 1.45)
Gender (Male vs. Female)			0.99 (0.81, 1.20)
Race			
Black			Ref
White			0.57 (0.46, 0.71)
Other Race			0.69 (0.52, 0.91)
AUC (95% CI)	0.87 (0.82, 0.91)	0.94 (0.91, 0.97)	0.95 (0.92, 0.98)

**Fig. 2:**
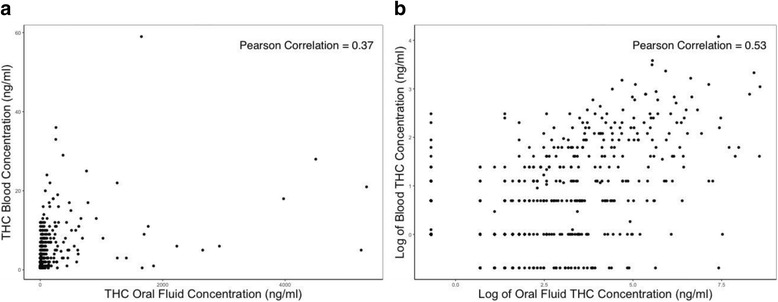
Scatter Plots of THC Concentrations in Blood and Oral Fluid: (a) Original Scale and (b) Log-transformation (replacing zero values with 0.5), among Driver with Non-zero Values in either Blood or Oral Fluid (*n* = 500)

Figure 2 shows the scatter plots and estimated Pearson correlation coefficients for continuous THC concentration in blood and in oral fluid on both the untransformed and log-transformed scales. Log-transformed values were associated with a higher Pearson correlation coefficient (0.53 vs. 0.37), indicating a stronger linear association and as such, we used these transformed values in linear regression models. The linear regression models examining the association between the natural-log blood THC concentration and the natural-log oral fluid THC concentration shows that a 10% increase in oral fluid THC concentrations was associated with a 2.4% (95% CI: 2.1%, 2.8%) increase in blood THC concentrations (Table 3). Drivers who used marijuana in the past 24 h had a higher blood THC concentration than drivers who never used marijuana or who last used marijuana a year or more prior to testing. We compared the point estimates and 95% confidence intervals of the coefficients in Tables 2 and 3 to those with multiple imputation and survey weighting adjustment and found that the results were consistent.

**Table 3:** Estimated Regression Coefficients (*β*) and 95% Confidence Intervals (CI) in Adjusted and Unadjusted Linear Regression Models of the Log-transformed Blood THC (ng/ml) on Log-transformed Oral Fluid THC (ng/ml) among Drivers with Non-zero THC Concentrations in either Blood or Oral Fluid, Continental United States, Nighttime Weekend and Daytime Friday, 2013–2014

	Model 1 (n = 500)	Model 2 (*n* = 478)	Model 3 (*n* = 472)
	*β* (95%CI)	*β* (95%CI)	*β* (95%CI)
Intercept	0.14 (0.01, 0.26)	0.00 (−0.17, 0.17)	−0.01 (−0.31, 0.28)
Log of Oral Fluid THC Concentration (ng/ml)	0.25 (0.22, 0.29)	0.25 (0.21, 0.29)	0.25 (0.22, 0.29)
Blood Alcohol Concentration (ng/dl)			
0		Ref	Ref
1–39		− 0.29 (− 0.77, 0.19)	− 0.22 (− 0.72, 0.28)
40+		− 0.18 (− 0.60, 0.25)	− 0.15 (− 0.58, 0.27)
Last time use of Marijuana			
Over a year/Never		Ref	Ref
Over a Month		0.15 (−0.13, 0.42)	0.16 (−0.12, 0.44)
Past 2 days to a month		0.22 (−0.01, 0.45)	0.22 (−0.01, 0.46)
Past 24 Hours		0.29 (0.08, 0.49)	0.30 (0.10, 0.51)
Age (years)			
16–20			Ref
21–34			0.05 (−0.17, 0.27)
35–54			0.06 (−0.20, 0.33)
≥ 55			−0.13 (− 0.56, 0.30)
Gender (Male vs. Female)			−0.12 (− 0.29, 0.06)
Race			
Black			Ref
White			−0.03 (−0.21, 0.15)
Other Race			0.27 (0.01, 0.52)
R^2^	0.29	0.30	0.31

## Discussion

This study showed that oral fluid tests are reasonably accurate in detecting blood THC positivity with a 79.4% of sensitivity and 98.3% of specificity. The sensitivity and specificity are similar across subgroups of drivers with different characteristics, except for drivers older than 55 years of age (a low sensitivity of 54.6%) and drivers who reported using marijuana in the past 24 h (a low specificity of 66.7%). Positivity in oral fluid THC is strongly associated with positivity in blood THC (OR = 11.36). Information on BAC and last time use of marijuana may be used to enhance our ability in predicting the positivity in blood THC. These results suggest that the oral fluid test is a valid method for detecting the presence of THC in the blood. Our study reaffirms the findings reported by Kelley-Baker et al. (2016).

There exists a fairly strong linear relationship between the log-transformed oral fluid THC concentrations and the log-transformed blood THC concentrations (Pearson correlation coefficient = 0.53, *p* = < 0.001). This finding adds to the existent evidence that oral fluid THC is associated with blood THC (Langel et al. 2014; Gjerde et al. 2014). We improved the prediction models by including covariates and using the log-transformation to yield a better linear correlation. However, given that oral fluid THC concentration only explained 29% of variation in the blood THC concentration, it is not advisable to derive blood THC concentrations from oral fluid THC concentrations.

We defined blood THC positivity using the detection limit (≥ 1 or > 0 ng/ml) as the cut-off. Alternatively, we conducted a sensitivity analysis by using other cut-off points with > 1, 2, 3, and 5 ng/ml. Because there was no driver with a blood THC concentration of 1 ng/mL in the study sample, the sensitivity-specificity analysis using > 1 ng/ml as the cut-point yielded the same results as that using > 0 ng/ml. When a cut-point of > 2 ng/ml was used, the sensitivity improves significantly from 79.4% to 91.7% but the specificity reduces from 98.3% to 95.3% when the positivity in the oral fluid sample was defined as > 0 ng/ml. This sensitivity analysis supports our conclusion that the oral fluid test is a valid alternative to detect the positivity of THC in the blood. However, given that many states have adopted a zero-tolerance policy toward drugged driving, the analysis using the cut-off point of > 0 ng/ml to define blood THC positivity provides the most relevant results for assessing the validity of using oral fluid test as a screening tool for detecting marijuana-impaired DUI.

We then identified optimal cut-off concentration in oral fluid that provided the highest possible diagnostic accuracy for predicting positivity of THC in blood using accuracy and AUC. We found that a cut-off value of ≥ 2 and ≥ 3 ng/ml in oral fluid achieved the best diagnostic accuracy. We chose to use the cut-off value of ≥ 2 ng/ml (same as using > 0 ng/ml) because it is also the minimum detectable level in the oral fluid sample. This is consistent with other studies using a cut-off value of ≥ 2 ng/ml (Kelley-Baker et al. 2016; Verstraete 2005), although a cut-off of 38 ng/ml was used in a population with low prevalence of drugs use (Gjerde et al. 2014).

It is worth noting that less than half of the eligible drivers (41.4%) consented to provide both whole blood and oral fluid samples and these consenting drivers were less likely to be white and report using marijuana recently than those who refused to provide specimens. Therefore, our study results are potentially susceptible to bias. Driver age, race and last time use of marijuana were reported by the drivers via electronic tablet and thus can also be subject to bias. Moreover, the measurement of blood and oral fluid THC concentrations was limited by the detection limits of ELISA and LC/MS/MS screening and confirmation tools. With the improvement of the detection limits in both oral fluid and whole blood samples in the future, we expect to observe a higher degree of agreement in the positivity of THC tests between the oral fluid and blood samples. Although THC is the most important psychoactive constituent of cannabis, there are other chemical compounds in marijuana that may impair driving safety. Currently, marijuana test based on oral fluids is limited primarily to detecting THC only.

Notwithstanding, the 2013 NRS provided valuable data in assessing the validity of the oral fluid test as a less invasive and less costly alternative to blood test in detecting the presence of THC in the blood. Further research is needed to better understand the relationship between blood and oral fluid THC concentrations and to develop a more reliable algorithm for calculating blood THC concentrations based on oral fluid testing results.

## Conclusions

Oral fluid test is a highly valid method for detecting the presence of THC in the blood and thus could be used as a screening tool for detecting marijuana-related DUI. Although there is a significant linear association between the log-transformed blood THC concentrations and the log-transformed oral fluid THC concentrations, log-transformed oral fluid THC concentrations can explain only 29% of the variation in log-transformed blood THC concentrations. Therefore, the oral fluid test is not an accurate method for estimating THC concentrations in the blood.

## Abbreviations

AUC: Area under receiver operating characteristic curve
BAC: Blood alcohol concentration
CI: Confidence interval
DUI: Driving under the influence
ELISA: Enzyme-linked Immunosorbent Assay
LC/MS/MS: Liquid/gas chromatography and mass spectrometry
ng/ml: Nanograms per milliliter
NRS: National Roadside Survey
OR: Odds ratio
THC: Delta 9-tetrahydrocannabinol
